# Variations in statin prescribing for primary cardiovascular disease prevention: cross-sectional analysis

**DOI:** 10.1186/1472-6963-14-414

**Published:** 2014-09-20

**Authors:** Robert Fleetcroft, Peter Schofield, Mark Ashworth

**Affiliations:** Department of Population Health and Primary Care, Norwich Medical School, University of East Anglia, Norwich, NR4 7TJ UK; Department of Primary Care & Public Health Sciences, King’s College London, 9th Floor, Capital House, 42 Weston Street, London, SE1 3QD UK

**Keywords:** Hydroxymethylglutaryl-CoA reductase inhibitors, Primary health care, Cardiovascular diseases

## Abstract

**Background:**

Statins are an important intervention for primary and secondary cardiovascular disease (CVD) prevention. We aimed to establish the variation in primary preventive treatment for CVD with statins in the English population.

**Methods:**

Cross sectional analyses of 6155 English primary care practices with 40,017,963 patients in 2006/7. Linear regression was used to model prescribing rates of statins for primary CVD prevention as a function of IMD (index of multiple deprivation) quintile, proportion of population from an ethnic minority, and age over 65 years. Defined Daily Doses (DDD) were used to calculate the numbers of patients receiving a statin. Statin prescriptions were allocated to primary and secondary prevention based on the prevalence of CVD and stroke.

**Results:**

We estimated that 10.5% (s.d.3.7%) of the registered population were dispensed a statin for any indication and that 6.3% (s.d. 3.0%) received a statin for primary CVD prevention. The regression model explained 21.2% of the variation in estimates of prescribing for primary prevention. Practices with higher prevalence of hypertension (β co-efficient 0.299 p <0.001) and diabetes (β co-efficient 0.566 p < 0.001) prescribed more statins for primary prevention. Practices with higher levels of ethnicity (β co-efficient-0.026 p <0.001), greater deprivation (β co-efficient −0.152 p < 0.001) older patients (β co-efficient −0.032 p 0.002), larger lists (β co-efficient −0.085, p < 0.001) and were more rural (β co-efficient −0.121, p0.026) prescribed fewer statins. In a small proportion of practices (0.5%) estimated prescribing rates for statins were so low that insufficient prescriptions were issued to meet the predicted secondary prevention requirements of their registered population.

**Conclusions:**

Absolute estimated prescribing rates for primary prevention of CVD were 6.3% of the population. There was evidence of social inequalities in statin prescribing for primary prevention. These findings support the recent introduction of a financial incentive for primary prevention of CVD in England.

**Electronic supplementary material:**

The online version of this article (doi:10.1186/1472-6963-14-414) contains supplementary material, which is available to authorized users.

## Background

Cardiovascular disease (CVD) is the leading cause of death in most developed countries, including the United Kingdom (UK) [1]. Statins are effective in reducing mortality and morbidity in patients with established coronary heart disease and stroke (secondary prevention), and also in people with higher levels of risk factors who have not yet developed clinically manifest CVD (primary prevention) [2]. The use of statins in both primary and secondary prevention is supported by guidelines internationally [3–6]. Until recently, most guidelines recommended treatment for primary prevention for those with a 20% or greater 10-year risk of developing CVD. The high risk population for primary prevention includes most of the elderly population, those with diabetes, and many with hypertension. Recently the threshold of 10 year CVD risk for primary prevention treatment has been lowered to ≥7.5% in the USA, and ≥10% in England with all patients over the age of 85 recommended the intervention [7, 8].

In the England there are substantial financial incentives for general practitioners (GPs) which encourage the use of statins for secondary prevention of CVD, whereas until recently the use of statins in primary prevention was only incentivised for patients diabetes [9]. In 2010, England introduced a financial incentive for CVD risk calculation in newly diagnosed hypertensive patients, and also NHS Health Checks to people between the ages of 40 and 74 years [10]. Both measures are likely to have increased awareness of the need for treatment with statins.

There is widespread evidence for variation in the provision of treatment for secondary prevention of CVD, including lower rates of statin prescribing in some ethnic minority groups, the elderly, and those from lower socioeconomic classes [11–16]. However for the case of primary prevention of CVD there is limited research into variations in prescribing rates of statins, possibly because there is no routinely collected data for this prescribing indication. There are consistent findings from several small studies that prescribing rates are lower than expected when guideline recommendations are taken into account, including the Netherlands, Norway, Canada, Australia and England [17–22]. One study based in 12 European countries in 2006/7 reported that less than half of those eligible for a statin for primary prevention received a prescription [23]. One of the major goals of the Quality and Outcomes Framework (QOF) is to reduce social inequalities in health [24]. The QOF is a pay- for-performance contract introduced in England and the UK in 2004, financially rewarding primary care practices for performance in clinical, organisational, patient experience and additional services [24]. Our aim was to conduct a study using modelling of national routinely collected primary care data to estimate the variation in the rate of statin prescribing for primary CVD prevention. We also aimed to determine the role of possible determinants of health inequalities such as age, ethnicity and social deprivation.

## Methods

### Design

We performed a cross-sectional study with analysis at the practice level. The study population included all general practices in England with data available in 2006/7. We used a definition of primary prevention which includes diabetes and hypertension, unless there is coexistent heart disease or cerebrovascular disease [25].

### Ethical approval

This study was granted ethical approval by the Faculty of Medicine and Health sciences Research Ethics Committee, Norwich Medical School, (reference 209/10-1066).

### Prescribing data

We obtained prescribing data for statins from the ‘Prescription and Pricings Division’ of the NHS Business Authority for all practices in England in 2006/7. Prescribing volume data are calculated from prescriptions issued in primary care and dispensed by a pharmacist or dispensing surgery. These data were standardised based on the ‘defined daily doses’ (DDD) for each of the five statin drugs (atorvastatin, fluvastatin, pravasatatin, rosuvastatin, simvastatin) plus one combination drug (simvastatin with ezetimibe). The DDD is an international measure of prescribing developed by the World Health Organization and is the assumed average maintenance dose per day for a drug used for its main indication in adults [26].

### Practice and population characteristics data

We collected on practice and population characteristics for all practices in England for the year 2006/7 except where otherwise specified. We obtained data on the practice characteristics known to influence prescribing patterns from the General Medical Services database [27]. We used data on ethnicity, deprivation status and the percentage of the practice population over 65 years of age as indicators sensitive to social inequalities. We obtained ethnicity data from the 2001 census for the geographical location (Lower Layer Super Output Area, LLSOA) of each general practice. We combined the data for South Asian, Black and Chinese groups to calculate a practice level prevalence of ethnic minorities. We used IMD quintiles of social deprivation based on LLSOAs in 2010 [28]. We obtained data on the prevalence of CVD, stroke (including TIA), hypertension and diabetes from the Information Centre for Health and Social Care [29].

### Construction of framework to estimate prescribing rates for primary prevention

As data on prescribing rates for statins for primary prevention are not routinely collected for English primary care we constructed a model in order to estimate the volume of statins prescribed for primary CVD prevention. A worked example is displayed in Additional file 1. Firstly, we calculated the number of patients receiving a statin for any indication by adding together the statin DDDs and dividing the total by 365 to estimate the number of patients receiving a statin over a year. Secondly, we multiplied this value by 1.25 to adjust for adherence, based on a study of the size of the discrepancy between statin prescribing for both primary and secondary prevention, and dispensing of those prescriptions [30]. Thirdly, we estimated the volume of statin prescribing in secondary prevention based on the numbers of patients known to have CVD or stroke, and assuming that all patients with established CVD would be prescribed a statin. Fourthly, we adjusted the estimated value for statin prescribing to account for comorbidity (as some patients will have had both CVD and a stroke) using a method previously described [31]. This method uses prevalence data from QOF for each practice to estimate the proportion of the population who have a comorbidity, in order to avoid double counting of individuals who have a diagnosis for both CVD and stroke. Finally, in order to estimate the volume of statins prescribed for primary prevention, we deducted the predicted numbers of patients eligible for secondary prevention with statins from the total numbers of patients receiving a statin. We expressed this number as a percentage of the practice population.

This model makes the following four assumptions. First that all patients with CHD and stroke would be prescribed a statin as recommended in current guidelines internationally; second that the dose of statin used is the same as the ‘defined daily dose’; third that the adjustment for collection of statin prescriptions is transferable from the US to the English setting; and fourth that the co-morbidity correction factor is an accurate estimate of true co-morbidity. We tested these assumptions in sensitivity analyses.

### Statistical tests

We excluded practices from the analysis on the basis of their list size (<1000 registered patients) as very small practices are likely to be atypical. We conducted descriptive analyses and linear regression modelling using SPSS v18 (Chicago: SPSS Inc.). The outcome variable was the estimated percentage of the practice population who were treated with statins for primary prevention. The main explanatory variables were 1) the percentage of the practice population from an ethic minority, 2) the percentage of the practice population aged >65 years, and 3) the IMD quintile attributed to the practice. Other potential explanatory variables were practice list size, number of patients for each GP in the practice, whether the practice had postgraduate GP training status, the rural/ urban mix of the practice population, the percentage prevalence of diabetes, and the percentage prevalence of hypertension. Prevalence of diabetes and hypertension were included as treatment with statins is often indicated in these conditions. We tested for multicollinearity by examining the variance inflation factor (VIF) for each explanatory variable in the model.

### Sensitivity analyses

We performed five sensitivity analyses to test whether our model was robust to changes in the four assumptions contained in our model. First we assumed only patients with heart disease and stroke who had a cholesterol < 5 mmol/l were taking a statin, as most patients taking a statin will have a cholesterol lower than 5 mmol/l [32]; second we assumed the average dose of statin prescribed was the ‘average daily quantity’; third we assumed that only 70% of prescriptions were dispensed; fourth we assumed that only 60% of prescriptions were dispensed; fifth we assumed that no patients had a comorbidity of CHD and stroke. These results are displayed in Additional file 2.

## Results

Full data were available for 6155 of the 8192 (75.3%) English general practices, with 40,017,963 patients. Values for the estimates of prescribing of statins for primary prevention were normally distributed. Visual inspection of the scatterplots suggested a linear relationship between the dependent variable and the percentage of population from an ethnic minority, quintile of IMD and the percentage of population over 65 years. There did not appear to be influential outliers. Table 1 displays the descriptive statistics for the final regression model. An estimated mean of 10.5% (s.d.3.7%) of the practice population were prescribed a statin for any indication, and 6.3% (s.d. 3.0) were prescribed a statin for primary prevention.

**Table 1:** **Descriptive statistics, practice characteristics for practices in England in 2006/2007**

	n	Mean	Standard deviation
Prevalence of hypertension	8182	12.53%	3.73%
Prevalence of diabetes	8182	3.73%	1.06%
Percentage from an ethnic minority	8090	10.89%	17.65%
Practice list size, measure in 1000s of patients	8182	6.50	3.96
IMD national quintile. Quintile 5 is most deprived (categorical data)	8182	2.56	n/a
Percentage of population >65 years	8182	15.01%	5.16%
Degree of rurality (Scale 1–8; 1 has highest population density)	8090	5.16	0.65

Table 2 displays the final models of the regression equation. The regression model explained 21.2% of the variation in estimates of prescribing a statin for primary prevention. The strongest predictors of prescribing rates of statins for primary prevention were the prevalence of diabetes and hypertension. For every 10% increase in the prevalence of diabetes (e.g. from 3.7% to 4.1%) there was approximately a 5% increase in the percentage of the population taking a statin for primary prevention (from 6.3% to 6.6%); and for every 10% increase in the prevalence of hypertension (e.g. from 12.5% to 13.7%) there was approximately a 3% increase in the percentage of the population taking a statin for primary prevention (from 6.3% to 6.5%). In a very small number of practices the rates of estimates of prescribing were so low that insufficient prescriptions were issued to meet the predicted needs of their secondary care population of patients on the CHD and Stroke registers (0.5% of practices).

**Table 2:** **Regression model showing relation between practice characteristics and the prescribing of statins for primary prevention**

R^2^ = 0.212 n = 6155	Standardized coefficients	Unstandardized coefficients (β)	95% CI for β	P value
Prevalence of hypertension	0.319	0.299	0.267 to 0.332	<0.001
Prevalence of diabetes	0.187	0.566	0.477 to 0.655	<0.001
Percentage from an ethnic minority	−0.139	−0.026	−0.031 to −0.020	<0.001
Practice list size, measure in 1000s of patients	−0.114	−0.085	−0.102 to −0.068	<0.001
IMD national quintile (quintile 5 is most deprived)	−0.076	−0.152	−0.203 to −0.102	<0.001
Percentage of population >65 years	−0.053	−0.032	−0.053 to −0.011	0.002
Degree of rurality (Scale 1–8; 1 has highest population density)	−0.026	−0.121	−0.228 to −0.014	0.026

Values are percentage difference (adjusted) in statins prescribed for primary prevention.

Tests using the variance inflation factor of predictor variables ranged from 1.1 to 2.4, indicating that multicollinearity was not a problem. None of the sensitivity analyses altered the main findings of the study with one exception. Reducing the estimate of prescriptions for statins dispensed by the chemist to 70% and to 60% meant that the predictor variable ‘percentage of population over 65 years’ was no longer statistically significant.

## Discussion

### Summary

This study derived estimates for rates of prescription of statins for primary prevention based on data derived from 75% of all general practices in England. We estimated that 10.5% of the registered population were dispensed a statin and that in 6.3%, primary CVD prevention was the indication for prescribing. There was evidence for social inequities with higher ethnicity, older persons and increasing social deprivation being significant predictors of reduced prescribing rates of statins for primary prevention. The prevalence of diabetes and hypertension were strong predictors of estimates of statin prescribing in primary prevention, and this provides face validity for the model. An unexpected and important finding was that in a small minority of practices the estimates of prescribing rates of statins were so low that there were insufficient doses to meet the predicted needs of their population of patients who had already had a stroke or CHD. This study used data from 2006–7. Since then guidelines have changed which have increased the indications for prescribing statins for primary prevention, including lowering the 10 year CVD risk threshold to 10% and also including patients with rheumatoid arthritis and chronic kidney disease.

### Strengths and limitations

This is the first large scale study estimating the prevalence of statin prescribing for primary prevention of CVD and primary prevention in an ethnic minority population. The entire English population with available data was sampled which minimised the possibility of selection bias. The prime limitation is that we did not have access to patient-level data which meant that we had to estimate the volume of statins used in primary CVD prevention. We used proxy measures for the role of social deprivation which may underestimate social inequalities [28]. There may be inaccuracies in the data that we have sourced on QOF such as incorrect Read coding in the medical record, though these are likely to be small. Prescribing data measures only those prescriptions that have been both prescribed and dispensed but we were unable to determine true consumption. Some patients may have purchased statins directly from the pharmacist (over-the-counter use) resulting in a small underestimate of our total prescribing volumes [30]. The assumption that all patients with CVD or stroke were taking a statin unless they had been exception reported by their practitioner is likely to be an overestimate (thus underestimating prescribing for primary prevention). This overestimate is likely to be small, as one English study reported high prescribing rates with 97% of patients with heart disease were recorded as taking a statin after a heart attack in 2004/5 [33]. Other authors have reported lower prescribing rates of statins and we have taken this into account in the sensitivity analysis [34]. It is possible (although unlikely) that all eligible patients were prescribed therapy for primary prevention and none were prescribed therapy for secondary prevention.

The adjustment for comorbidity is likely to be an overestimate as it included other comorbidities (thus overestimating prescribing volumes for primary prevention). We could not determine how many patients were receiving a statin for peripheral vascular disease. This is an observational study and as such it does not demonstrate causation, and because it is an ecological study associations at the practice level may not apply to individuals.

### Comparison with the existing literature

This is the largest study to estimate rates of prescribing of statins for primary prevention in general practice in England, and the first to examine the effect of ethnicity on primary prevention with statins. The estimates for prescribing rates for primary prevention are similar to another UK study based in 421 practices in 2008 [22]. An English study using individual patient data from 2009 reported that 74% of patients with establish CVD received a prescription for a statin, which is similar to the sensitivity analysis [34]. Other studies have reported lower prescribing rates in an elderly population and that prescribing rates are related to the prevalence of diabetes and hypertension [19, 22].

## Conclusions

First there was evidence for inequity with lower estimates of statin prescribing for primary prevention with respect to ethnicity, older persons and social deprivation. Second, the estimated levels of statin prescribing were insufficient to offer adequate primary CVD prevention in some practices. The reasons for low prescribing rates for statins are uncertain but may include downward prescribing cost pressures, the use of lower than recommended doses, patient factors, and the lack of a financial incentive at the time for primary prevention of CVD. In light of the findings of our study together with two smaller studies demonstrating under prescribing of statins in primary prevention of CVD, further research using individual patient level data targeting socially deprived groups, ethnic minority groups and elderly groups is indicated [19, 22].

## Electronic supplementary material

Additional file 1: **Computations for numbers of patients receiving a statin for primary prevention.** (DOCX 15 KB)

Additional file 2: **Sensitivity Analyses.** (DOCX 16 KB)

## Abbreviations

CVD: Cardiovascular disease
IMD: Index of multiple deprivation
UK: United Kingdom
USA: United Stated of America
NICE: National Institute for Health and Care Excellence
NHS: National Health Service
QOF: Quality and outcomes framework
DDD: Defined daily dose
LLSOA: Lower layer super output area
VIF: Variance inflation factor.
