# Supplementary material for: Variations in statin prescribing for primary cardiovascular disease prevention: cross-sectional analysis
Source: BMC Health Serv Res. 2014 Sep 20;14:414. doi: 10.1186/1472-6963-14-414 (PMC4263070; doi:10.1186/1472-6963-14-414)
Supplement: Supplementary file 1 — Additional file 1: Computations for numbers of patients receiving a statin for primary prevention. (DOCX 15 KB) [file 12913_2014_3516_MOESM1_ESM.docx]

### Additional file 1 – Computations for numbers of patients receiving a statin for primary prevention

1 Total defined daily doses of statins =

∑ DDDs (atorvastatin, fluvastatin pravasatatin, rosuvastatin, simvastatin, simvastatin + ezetimibe)

2 Total patients treated with a statin ∑ DDDs/365

3 Correction for adherence ∑ (DDDs/365) * 1.25

4 Numbers of patients treated for secondary prevention =

∑ (patients with heart disease) + ((patients with stroke) * comorbidity correction factor))

5 Numbers of patients treated for primary prevention =

Total numbers treated minus numbers treated for secondary prevention=

(∑(DDDs/365) * 1.25) – (∑ (patients with heart disease) + (patients with stroke * comorbidity correction factor))

6 Percentage of patients treated for primary prevention=

(Numbers of patients treated for primary prevention*100)/practice list size
