# Supplementary material for: Variations in statin prescribing for primary cardiovascular disease prevention: cross-sectional analysis
Source: BMC Health Serv Res. 2014 Sep 20;14:414. doi: 10.1186/1472-6963-14-414 (PMC4263070; doi:10.1186/1472-6963-14-414)
Supplement: Supplementary file 2 — Additional file 2: Sensitivity Analyses. (DOCX 16 KB) [file 12913_2014_3516_MOESM2_ESM.docx]

### Additional file 2 – Sensitivity Analyses

| **assumption** | **sensitivity analysis 1** | **Sensitivity analysis 2** | **Sensitivity analysis 3** | **Sensitivity analysis 4** | **Sensitivity analysis 5** |
| --- | --- | --- | --- | --- | --- |
| **definition** | **Patients with CHD and stroke with cholesterol < 5mmol/l taking statin** | **Typical dose taken was the ‘average daily quantity’** | **70% of prescriptions exchanged for statins at the chemist** | **60% of prescriptions exchanged for statins at the chemist** | **No comorbidity between patients with CHD and stroke** |
| ***Percentage from an ethnic minority** | **-2.57**  **(-2.99 to**  **-2.15)** | **-0.026**  **(-0.031 to -0.021)** | **-0.032**  **(-0.038 to**  **-0.036)** | **-0.041**  **(-0.048 to**  **-0.034)** | **-1.47**  **(-1.87 to -1.074)** |
| ***IMD national quintile** | **-17.03**  **(-21.10 to**  **-12.96)** | **-0.153**  **(-0.203 to -0.103)** | **-0.201**  **(-0.258 to**  **-0.026)** | **-0.265**  **(-0.330 to**  **-0.197)** | **-6.48**  **(-10.28 to -2.67)** |
| ***Percentage of population >65 years** | **-9.70**  **(-11.39 to**  **-8.00)** | **-0.029**  **(-0.050 to -0.008)** | **-0.017 (ns)**  **(-0.041 to 0.007)** | **0.002 (ns)**  **(-0.026 to**  **0.030)** | **-1.97**  **(-3.56 to -0.39)** |
| **Adjusted R^2^** | **0.517** | **0.213** | **0.239** | **0.267** | **0.513** |
| **% of practice population treated with a statin for primary prevention** | **6.30**  **(s.d. 3.25)** | **6.33 (s.d.3.01)** | **7.81**  **(s.d. 3.51)** | **9.80%**  **(s.d. 4.19)** | **5.85**  **(s.d. 2.96)** |

* β coefficients (95% CI)
